# The invisible life

**DOI:** 10.3389/fmicb.2024.1401487

**Published:** 2024-05-20

**Authors:** Francesca Guerrieri, Cédric Libert

**Affiliations:** ^1^Cancer Research Center of Lyon (CRCL), UMR Inserm 1052 - CNRS 5286, Lyon, France; ^2^Ecole Nationale Superieure d'Architecture de Saint-Etienne, Saint-Etienne, France

**Keywords:** microbiota, urbanism, architecture, sequencing, city

## Introduction

With the advent of next-generation sequencing (NGS), some scientific assumptions have changed. The multiple sequencing of the bacterial genome revealed that almost all areas of the human body are inhabited by microbial communities, collectively known as the microbiota. Primarily, it has become clear that many processes previously attributed to the human body alone are, in fact, the result of interactions between the human body and the bacteria that inhabit it. The human being is, therefore, an ecosystem itself. While this has by now become a premise of much scientific research, both basic and translational, the profound implications of this notion for the interplay between the environment and humans are less clear (Yatsunenko et al., 2012). Our homes, our cities, are not empty (Gilbert and Stephens, 2018; Guerrieri, 2022). They, too, are ecosystems, which include bacterial communities that interact with us. A red thread of microorganisms inextricably links our existence with the place we live in. Thus, the boundaries between what is internal and what is external, between self and non-self, are blurred. The home, for example, archetype of the self, a symbol of our identity, of the unconscious, designed based on organic projection and isolation from the environment, must then be reimagined as perfectly connected with its surroundings. The buildings around us are covered with microbial communities that give them an identity beyond design and color, which contribute to shaping their future and their function. Microbes in the built environment can also influence the health of a place, as highlighted by several studies (Mahnert et al., 2019; Sun et al., 2023). However, if this point can be easily understood, the breakthrough was understanding that the environmental microbiota has not only a precise identity linked to the geographical position but also to the habits and culture of those who live there. Recent literature revealed that the presence of humans can influence the microbial core of a space, such as our apartments (Lax et al., 2014; Xie et al., 2023) or our cities (McCall et al., 2020). Thus, these invisible communities living inside and outside of us change with us, and we change with them (Kang et al., 2018). We modify spaces not only by furnishing them but also by colonizing them with our microbiota. A recent study carried out in Germany, took into account more than 8,000 people and more than 2,000 families with numerous etiological and cultural factors, put forward an important conclusion that microbiota is mainly associated with cohabitation and the environment rather than genetic relationship (Gacesa et al., 2022). So, *I live in a place, therefore I am*, to paraphrase Descartes. In addition, we live in the cities. The percentages are clear: nowadays, 50% of the world's population lives in cities, and these percentages are expected to reach 70% within a few years. Urbanization is the phenomenon that characterizes contemporary society (https://population.un.org/wpp/publications/files/key_findings_wpp_2015.pdf). The first global Atlas of urban microbiota, including buildings and the public transport, was published in 2021. It states that the worldwide consortium MetaSUB (founded in New York City in 2018 and already ongoing) collected and sequenced the microbiome in 60 cities around the world. Cities, regardless of the country of origin in which they are located, have a similar microbial profile for 85% of bacteria: a fingerprint of microorganisms that we could define as “urban” (Danko et al., 2021), that, also if rich, seems to be characterized by less bacterial heterogeneity, more pathogens, and more antibiotic-resistant bacteria (Chen et al., 2023). Meanwhile, we can distinguish the microbiota of the person who lives in the city as *the urban gut* (Ayeni et al., 2018; Jha et al., 2018; Sonnenburg and Sonnenburg, 2019; van der Vossen et al., 2023). It is remarkable that literature has shown that the *urban gut* is characterized by lower bacterial diversity and the incidence of microbiota-affected diseases [inflammatory bowel diseases, allergies, or antimicrobial resistance ((AMR)] of people living in the city is higher than the others in the countryside (Nicolaou et al., 2005; Zuo et al., 2018; Sonnenburg and Sonnenburg, 2019). Of note, the World Health Organization (WHO) has identified AMR as one of the most dangerous threats to humans and food safety. Diet, pollution, use of antibiotics, and excessive hygiene, which are the keywords of urbanism, affect our microbiota and arguably have an impact on our health (Claus et al., 2016; Schmidt, 2017; Zhai et al., 2018; Cavicchioli et al., 2019; Guerra et al., 2020; Soininen et al., 2022).

## Building with science

Thus, urbanization is increasing; the urban microbiota and the *urban gut* are less rich in biodiversity; and diseases linked to the microbiota are increasing. The loss of microbial diversity correlates with an increase in resistance, and, consequently, the need for implementing strategies to restore bacterial diversity along the growing urbanization became so mandatory for public health. But what we can do? The urban gut and especially the microbiota-linked diseases are the object of multiple studies, but what about the bacteria urban profile of the cities? Knowing that bacterial communities are modulated by the use of antibiotics and probiotics, could we speculate about the use of both to modify the urban microbiota of cities, for example?

Just as we use antibiotics or probiotics for ourselves, we can imagine doing it for cities. Unintentionally, hospitals have already partially answered one question. Scientific research has brought to light that sterilizing the hospital does not address the problem; removing harmless bacteria from a place may create an even more dangerous ecosystem, and the hospitals have been characterized by microbial dysbiosis and reservoirs of AMR (Cason et al., 2022). Sterilization and cleaning reduced bacterial diversity (Mahnert et al., 2019). Of note, if the hospitals are well-known places driving antimicrobial resistance dissemination in the urban environment, very little information is available about schools, offices, or public spaces. Around the world, several consortiums are trying to bring to light this subject. Among others, we are developing the MUSE project, a transdisciplinary collaborative project, that involves École Nationale Supérieure d Architecture de Saint-Étienne, France (ENSASE) and INSERM laboratory (Lyon, France) and seeks to characterize both the Saint Étienne urban microbiota fingerprint and its effect on the city's residents. By gathering and analyzing metagenomics data from both urban and human sources (skin and fecal samples), we aim to explore the relationship between humans and their environment. This will help us understand whether certain places, due to inherent or social characteristics, exhibit lower biodiversity and determine whether this is indicated by significant antibiotic resistance or the presence of certain phyla in a state of dysbiosis. Additionally, ENSASE plans to implement green space interventions in public spaces to influence the microbiota communities through temporary constructions and boost urban regeneration.

**Figure 1:**
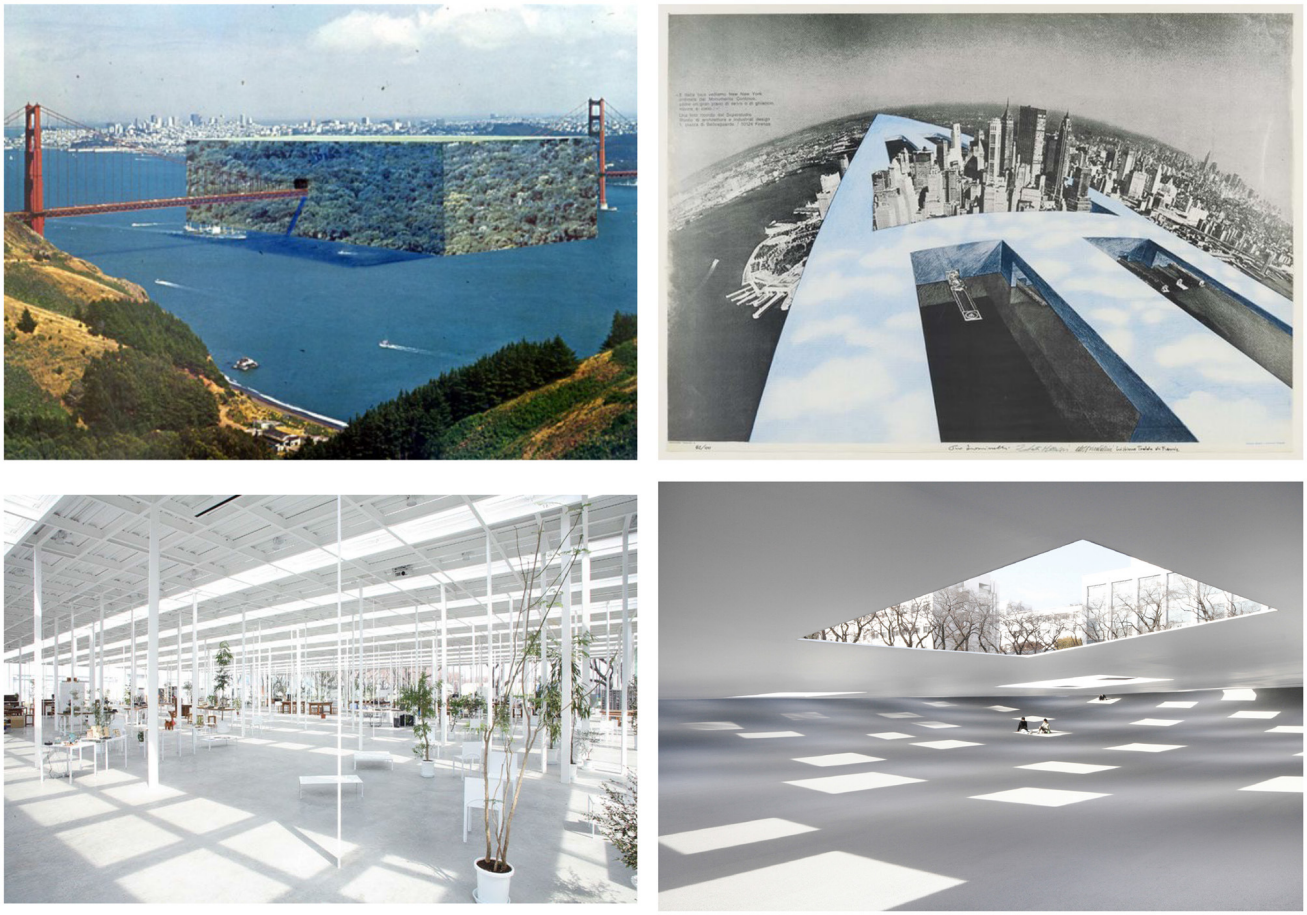
**(Upper panel)** The 12 ideal cities, Superstudio. **(Lower panel)** The Plaza of Kanagawa Institute of Technology. Junya Ishigami architect.

Recently, several groups rather tried to use probiotics to promote beneficial microbes (Caselli et al., 2016; D'Accolti et al., 2023; Leistner et al., 2023). However, using probiotics on building surfaces or the subway no longer works, because the city, because of how it is built, exerts a selective pressure on the microbiota. Indeed, architectural design exerts an influence on the indoor microbiome and urban space interventions that can determine health, wellbeing, and social effects (Berg et al., 2014; Bope et al., 2018; Robinson et al., 2018; Li et al., 2021). However, if the scientific community is only now starting to understand the essential importance of the microbiome in human health, architecture is also beginning to ask questions about the role of micro-living in space. Can the microbiota become an infrastructure? Architecture has always asked itself questions about how design can create a “healthy” place for human beings: from the first projects aimed at minimizing humidity, continuing to “the ideal city” by Le Corbusier, to the latest projects on biomaterials capable of orienting the microorganism's ability to settle in a place. From Superstudio's 12 ideal cities which pushed to the edge individual aspects of contemporary planning (zoning, uniformity, existence minimum, transparency, building industrialization, climatization, etc.), to the most recent *invisible* projects fused with the environment by Junya Ishigami, each aspect showed its insufficiency in the face of the problems connected with the complexity of the ever-changing city (Figure 1).

**Figure 2:**
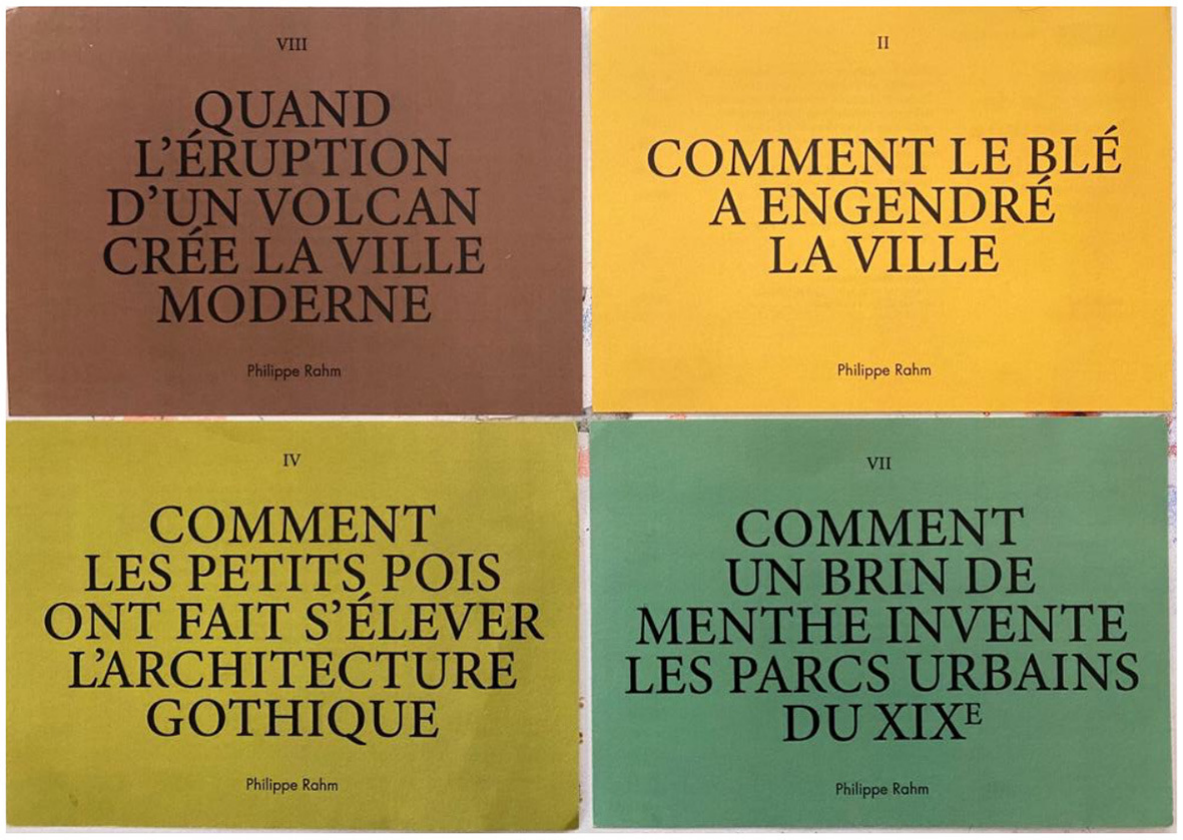
Panels of Philippe Rahm's exhibition on the rereading of architecture.

**Figure 3:**
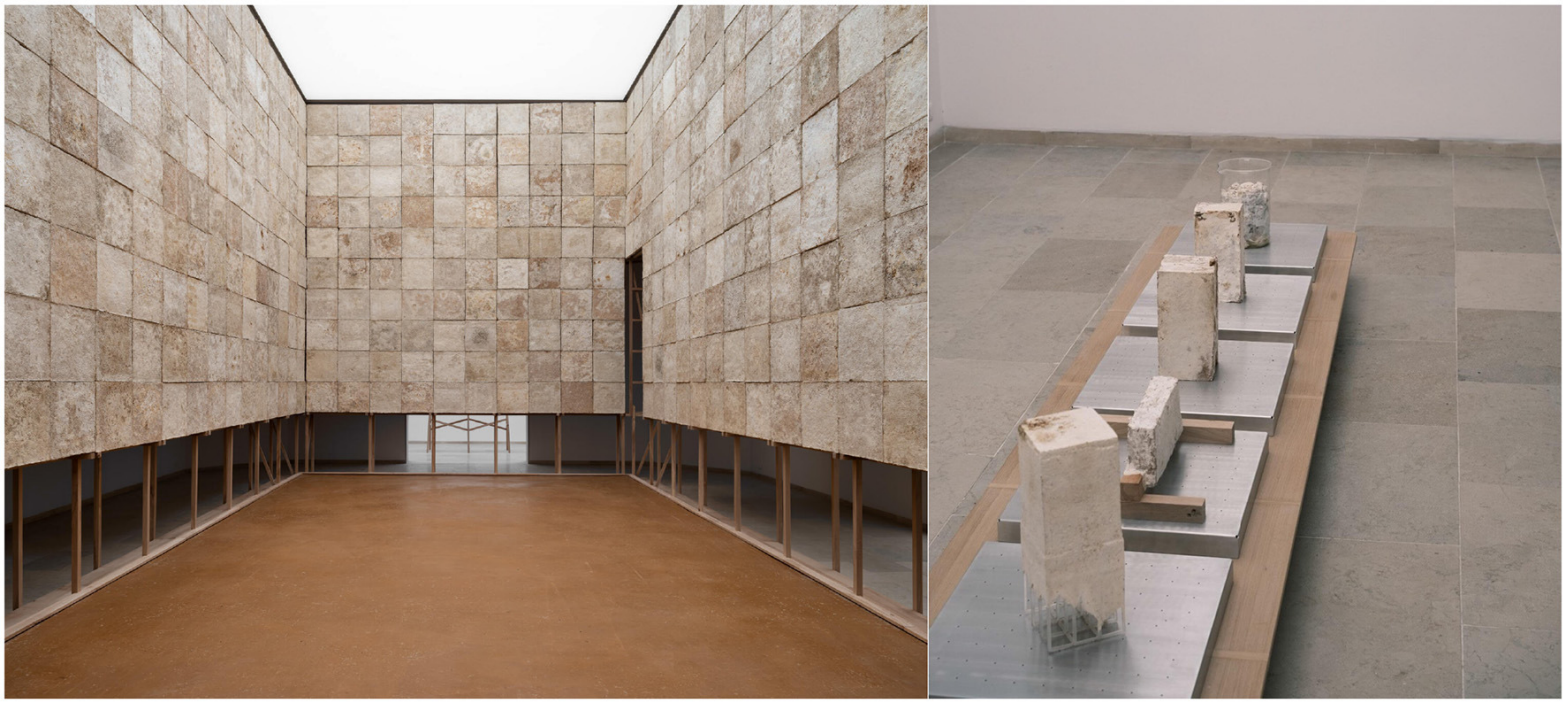
“*In vivo*” Belgian Pavilion, Venice Biennale 2023.

Recently, the architect P. Rahm in “*The Natural History of the Architecture*” considers the natural, physical, biological, or climatic causes that have influenced the course of architectural history and shaped urban forms over the centuries (Rahm, 2020). He proposed that rereading the history of architecture from these objectives, material, real data make it possible to face the major environmental challenges of our century (Figure 2). The discovery of microbial communities interacting with our built environment has far-reaching implications for the discipline of architecture and the design of cities. One can only imagine what microbial data would offer about the efficiency of the hygienist theories massively implemented all around the globe by the “Modern Movement” or regarding the sociopolitical distribution of healthy and unhealthy microbiota within the urban realm. These important questions support the idea of how important it is for microbiology and architecture to establish collaborations for a microbiome-informed design. In architecture, the description of our environment (built or natural) encompasses many forms, regularly convened over time to cover their unlimited diversity. A broad range of literature and iconography was explored throughout the history of architecture and urbanism (Venturi et al., 1978; Frampton, 1980; Clément, 1999; Le Corbusier, 2008; Banham, 2011; Morin, 2014; Libert, 2016; Ghyoot et al., 2018; Latour and Weibel, 2020; Thévenin, 2021). More recently, citizen participation was the center of all attention while in parallel appeared new critical and self-reflexive approaches to human sustainability related to climate change and other damages made to our environment—the present moment of the Anthropocene. Further ambitions toward the understanding of our (built) environment are now to be able to describe the reality of one world in light of its multiple behaviors. Indeed architecture and urbanism recently broadened their field of interest and research to non-visible and microbial life as equally part of our inhabited environment—the conception of a global ecosystem we are part of and belong to. This shift represents a serious paradigm shift toward the understanding of “Life on Earth.” The Belgian pavilion of the Biennial of Venice questions how to rethink architecture and titles the pavilion “*in vivo*” (Figure 3). The structure, that we can define as a “living” material, was built from and with fungi. Architects and academics at University College London created algae biopanels called “Indus” tiles that can absorb pollutants (https://www.ucl.ac.uk/bartlett/architecture/news/2019/apr/innovative-bio-integrateddesign-wins-water-futures-design-challenge) (Dubey et al., 2023). Urban algae-based innovations are rising, including the development of air-purifying booths and building façades containing algae, such as the Bio-IQ building in Hamburg. An intriguing project developed a novel ceramic for architectural setting, inoculated with *Bacillus subtilis*, a Gram-positive soil bacterium with several benefits, including promoting plant growth and enhancing gut health (Robinson et al., 2024).

To conclude, it is fundamental contemporary necessity—creating a bridge between architecture and biology: setting up a collaborative scope of research together with urban design assignments and public health politics, we can redefine the concept of cure and care.

## Author contributions

FG: Conceptualization, Data curation, Formal analysis, Funding acquisition, Investigation, Methodology, Project administration, Resources, Software, Supervision, Validation, Visualization, Writing – original draft, Writing – review & editing. CL: Conceptualization, Data curation, Formal analysis, Funding acquisition, Investigation, Methodology, Project administration, Resources, Software, Supervision, Validation, Visualization, Writing – original draft, Writing – review & editing.
